# Letter to the editor on: Oral care and nosocomial pneumonia: a systematic review. einstein (São Paulo). 2015;13(2):290-6.

**DOI:** 10.1590/S1679-45082015CE3478

**Published:** 2015

**Authors:** Leopoldo Penteado Nucci da Silva

**Affiliations:** Faculdade Estácio de Sá de Goiás, Goiânia, GO, Brazil.

To the Editor:

I read with interest the systematic review “Oral care and nosocomial pneumonia: a systematic review” published in einstein (São Paulo),^1^ which describes evidences of an association between oral care and reduction in the incidence of nosocomial pneumonia. I would like to express some concerns about the study. First, the search strategy used was not reported, therefore the search strategy did not allow replication. In addition, articles included and excluded in the study were not identified in the table. I conducted a search in PubMed/MEDLINE and LILACS using the uniterms mentioned by the authors of the review ((((((*nosocomial pneumonia*) *AND oral care*)) *OR* ((*nosocomial pneumonia*) *AND oral hygiene*)) *OR* ((*nosocomial pneumonia*) *AND oral microflora*))) *OR* (((((*pneumonia associated with mechanical ventilator*) *ANDoral care*)) *OR* ((*pneumonia associated with mechanical ventilator*) *AND oral hygiene*)) *OR* ((*pneumonia associated with mechanical ventilator*) *AND oral microflora*)). The search retrieved 235 reports in PubMed/MEDLINE and 20 reports in LILACS. Because the authors did not describe the number of articles retrieved in each database, I could not compare the results found. The authors identified 297 reports in 3 databases, and 52 had full-text available; of these, only 14 were included in the study. In my search I identified more full-text articles. I inquire about this difference presented in the flowchart. In addition, what criteria do authors used to include or exclude an article, when reviewers’ opinion was different? Was there a third independently and blinded author in charge to decide whether to exclude or include an article? Did an agreement exist between the two first reviewers? Perhaps, the answer to these questions can explain the inclusion of less articles in the sample.

Second, two recent studies^2,3^ included in the review reported that the association was unclear between the use of 0.12% chlorhexidine in oral care and reduction of nosocomial pneumonia. In addition, the second study^3^highlighted that the use of chlorhexidine in a specific group of patients is efficient, but because of methods divergences among the selected studies, independent evaluation was not possible in terms of the influence of oral care based on identification and risk control, which was performed by trained professional in assessment issues related with the stomatognathic system. Therefore, it is not only about oral hygiene or to create hygiene protocols, and the use or not of auxiliary chemical agents (chlorhexidine, methylene blue, ozone, etc.). The reduction in the incidence of nosocomial pneumonia is likely to improve with a multidisciplinary team working in which the oral surgeon, especially trained to and who is part of the routine of institutional team, help to improve oral hygiene process along with nursing team. In addition, the nursing team, when facing severe clinical situations, should contact the oral surgeon to evaluate and control a patient’s systemic decline using the treatment options currently available.

Sincerely,

Leopoldo Penteado Nucci da Silva Faculdade Estácio de Sá de Goiás, Goiânia, GO, Brazil.
