# Supplementary material for: Letter to the editor on: Oral care and nosocomial pneumonia: a systematic review. einstein (São Paulo). 2015;13(2):290-6
Source: Einstein (Sao Paulo). 2015 Jul-Sep;13(3):480. doi: 10.1590/S1679-45082015CE3478 (PMC4943803; doi:10.1590/S1679-45082015CE3478)
Supplement: Translation

# Translation: Carta ao editor para: Cuidados bucais e pneumonia nosocomial: revisão sistemática. einstein (São Paulo). 2015;13(2):290-6.

**Authors:** Leopoldo Penteado Nucci da Silva

**Affiliations:** Faculdade Estácio de Sá de Goiás, Goiânia, GO, Brasil.

Editor:

O artigo de revisão “Cuidados bucais e pneumonia nosocomial: revisão sistemática”, publicado na einstein (São Paulo),^1^ apresenta uma série de evidências da associação entre higienização bucal e a diminuição da incidência de pneumonia nosocomial. Com relação ao método de busca das publicações, não foi informada a estratégia realizada e, por ser uma revisão sistemática, ficou inviável a realização de uma atualização fechada. Como também não apresentou a tabela de identificação dos artigos aceitos ou rejeitados, refiz uma estratégia de busca utilizando os unitermos citados, ((((((*nosocomial pneumonia*) *AND oral care*)) *OR* ((*nosocomial pneumonia*) *AND oral hygiene*)) *OR* ((*nosocomial pneumonia*) *AND oral microflora*))) *OR* (((((*pneumonia associated with mechanical ventilator*) *ANDoral care*)) *OR* ((*pneumonia associated with mechanical ventilator*) *AND oral hygiene*)) *OR* ((*pneumonia associated with mechanical ventilator*) *AND oral microflora*)), nas bases de dados PubMed/MEDLINE e LILACS. Foram identificados 235 artigos na base de dados PubMed/MEDLINE e 20 na LILACS. Como os autores não informaram o número de artigos recuperados por base de dados, não foi possível qualquer comparação entre os diferentes momentos de busca. Já em relação ao processo de inclusão, os autores identificaram inicialmente 297 artigos em 3 bases, sendo que somente 52 artigos estavam disponíveis na íntegra; destes, somente 14 foram selecionados para a amostra do estudo. No processo de consolidação da nova amostra citada no item anterior, mais artigos estão na íntegra, o que cria uma discrepância em relação ao fluxograma descrito. Mesmo utilizando dois pesquisadores cegos, quando há divergência entre eles, os autores incluíam ou excluíam o artigo? Um terceiro autor independente e também cego de autoria realizava esse procedimento? Ou havia dominância entre os dois pesquisadores que executaram esse procedimento? O que justificaria o número menor de artigos incluídos na amostra deste estudo.

No entanto, com relação aos resultados, há dois estudos recentes^2,3^ que mostram que não é clara a associação entre o uso de clorexidina 0,12% nos cuidados bucais e a redução de pneumonia nosocomial. O segundo estudo^3^ até enfatiza que o uso da clorexidina em determinados grupos de pacientes é eficiente, mas, por divergências metodológicas entre os artigos selecionados, não há como avaliar independentemente a influência do cuidado na higienização bucal, por meio da identificação e do contingenciamento do risco, realizado por um profissional devidamente capacitado em avaliar e tratar questões inerentes ao sistema estomatognático. Portanto, não é somente higienizar ou criar protocolos de higienização, utilizando ou não agentes químicos auxiliares (clorexidina, azul de metileno, ozônio etc.); a redução do acometimento de pneumonia nosocomial está mais aderente à incorporação de grupos multiassistenciais, em que o cirurgião dentista, devidamente capacitado e incluso à rotina da equipe institucional, colabora com a melhoria dos processos de higienização bucal com a equipe de enfermagem, e esta, diante de situações clínicas de agravo, aciona o cirurgião dentista para avaliar e contingenciar o declínio sistêmico do paciente, por meio do arsenal terapêutico à disposição.

Atenciosamente,

Leopoldo Penteado Nucci da Silva Faculdade Estácio de Sá de Goiás, Goiânia, GO, Brasil.
